# Development and application of machine learning models for hematological disease diagnosis using routine laboratory parameters: a user-friendly diagnostic platform

**DOI:** 10.3389/fmed.2025.1605868

**Published:** 2025-10-01

**Authors:** Jingya Liu, Yang Gou, Wuchen Yang, Hao Wang, Jing Zhang, Shengwang Wu, Siheng Liu, Tinglu Tao, Yongjie Tang, Cheng Yang, Siyin Chen, Ping Wang, Yimei Feng, Cheng Zhang, Shuiqing Liu, Xiangui Peng, Xi Zhang

**Affiliations:** ^1^Medical Center of Hematology, The Second Affiliated Hospital of Army Medical University, Chongqing, China; ^2^State Key Laboratory of Trauma, Burns and Combined Injury, Jinfeng Laboratory, Chongqing, China; ^3^Chongqing Key Laboratory of Hematology and Microenvironment, Chongqing, China

**Keywords:** hematological diseases, machine learning, prediction model, SHAP, laboratory parameters, diagnostic platform

## Abstract

**Aim:**

In recent years, with the change of social environment, the incidence and detection rate of hematological diseases have shown an increasing trend. Early diagnosis and detection of hematological diseases are very important to improve the quality of life and prognosis of patients.

**Methods:**

In this study, we employed 54 clinical and conventional laboratory parameters. By optimally combining multiple feature selection methods and machine learning algorithms, we developed 7 machine learning models with varying feature set sizes. We comprehensively evaluated the performance of these models, analyzed the interpretability of the optimal and simplified models using SHapley Additive exPlanations (SHAP), and compared these two models with the diagnostic performance of hematologists. Finally, we developed a user-friendly diagnostic platform.

**Results:**

The results showed that the ensemble model_1 with 46 feature parameters (EnMod1-46) and the simple ensemble model_2 with 12 feature parameters (EnMod2-12) demonstrated significant performance in diagnosing 16 types of hematological diseases. On the temporally distinct test set_1, the EnMod1-46 achieved an accuracy of 0.804 and an area under the curve (AUC) of 0.964, while EnMod2-12 attained an accuracy of 0.784 and an AUC of 0.961. To further validate the model’s generalization performance, EnMod1-46 achieved an accuracy of 0.738 and an AUC of 0.973 on the independent external test set_2, while EnMod2-12 yielded an accuracy of 0.705 and an AUC of 0.962. SHAP analysis showed that PLT, WBC, MCV, HGB, RBC and age were significant parameters in both models. Comparative analysis of clinical diagnosis revealed that the performance of EnMod1-46 and EnMod2-12 outperformed junior hematologists, while EnMod1-46 was comparable to senior hematologists. Concurrently, based on EnMod2-12, we developed a user-friendly diagnostic platform to facilitate risk assessment and improve access to accurate diagnosis.

**Conclusion:**

This study provides an efficient and accurate screening method for hematological diseases, especially in resource-limited countries and regions.

## 1 Introduction

Hematological diseases include a wide range of benign and malignant disorders that affect the blood, bone marrow and lymphatic system. In recent years, with the change of social environment, especially the aging of population and the continuous development of medical technology, the incidence and detection rate of hematological diseases have shown an increasing trend (1). However, due to the strong heterogeneity of laboratory indicators in hematological diseases (2), diverse clinical manifestations, and frequent overlap with other systemic diseases, some patients have difficulty in obtaining timely and accurate diagnosis at the first visit. This often results in multiple medical consultations, increasing both patients’ suffering and the waste of medical resources. If benign hematological diseases are not detected early or managed properly, these conditions can lead to serious symptoms and complications that can significantly affect the quality of life for patients. According to the global cancer statistics from GLOBOCAN 2022, malignant hematological diseases such as non-Hodgkin lymphoma and leukemia exhibited high incidence and mortality rates worldwide (3). Modern treatment methods, such as targeted therapy, and hematopoietic stem cell transplantation, have significantly improved the prognosis of patients and increased the survival rate of patients (4, 5). However, misdiagnosis or delayed diagnosis in the early stages can significantly compromise patient’s prognosis (6).

Machine learning (ML) as a subset of artificial intelligence has been explored in various domains of hematological diagnosis, including laboratory testing, histopathology, flow cytometry and molecular data (7). Moreover, the model built using ML can accurately diagnose various types of hematological diseases and identify key parameters (8, 9). In clinical practice, easily accessible laboratory parameters have been proved to be an important data source for implementing disease diagnosis models. For example, Erler et al. improved the classification accuracy of thalassemia by building a backpropagation artificial neural network using erythrocyte related parameters such as red blood cell count and average red blood cell volume, with sensitivity and specificity both reaching 94.2% (10). With the employment of random forest algorithms, Guncar et al. developed models with 181 and 61 parameters to classify 43 hematological diseases. The accuracy for diagnosing the most likely hematological disease was 59% and 57%, respectively, while the accuracy for predicting the top five diagnoses improved to 88% and 86% (11). Park et al. further developed an ensemble model based on deep neural networks, using 88 different parameters to classify 39 hematological disorders, achieving accuracies of 65% and 93% for predicting the most likely single and top five diagnoses, respectively (12). These models achieved accurate prediction of hematologic diseases. However, some of them involve a large number of parameters, which complicates model training and computation. Moreover, the prediction accuracy for the most likely single disease remains relatively low. While other studies have reported accuracy above 0.90, these have generally focused on predicting or differentiating between specific diseases, such as certain types of leukemia (13, 14).

Therefore, developing highly accurate and computationally efficient prediction models and tools for common hematological diseases are significant in aiding clinical diagnosis and decision-making. In this study, a total of 16 types of hematological diseases and a group of healthy individuals were included. The disease types cover about 80% of hematological diseases, which have a high prevalence. We developed a prediction model, named EnMod1-46, which was an ensemble model trained using 46 parameters selected by random forest combined with recursive feature elimination with cross-validation (RF-RFECV) and achieved a high classification accuracy. In order to facilitate clinical application, we simultaneously constructed a simplified model, EnMod2-12, which utilized an ensemble of four ML algorithms trained on 12 routine complete blood count (CBC) parameters. This model also supported a practical online prediction tool, facilitating real-world application. This study provided valuable decision-making support for clinical practice and helped to realize early diagnosis and screening of hematological diseases.

## 2 Materials and methods

### 2.1 Participants

This study included 10,401 patients newly diagnosed at the Medical Center of Hematology of the Second Affiliated Hospital of Army Medical University from January 2015 to March 2024. The cohort comprised 16 groups of patients with newly diagnosed hematological diseases and a group of healthy individuals as controls (including healthy donors and healthy medical examination patients). Malignant hematological diseases include ALL, APL, AML-nonAPL, CLL, CML, CMML, MDS, MM, MPN, and Lymphoma. Benign hematological diseases include Hemocytopenia, AA, Thalassemia, MgA, HA, and IDA. The full names of the disease types are provided in the Glossary. Additionally, 342 patients from April 2024 to June 2024 were collected as the temporally distinct test set_1. The multi-center and independent external test set_2 included 149 patients recruited from two tertiary medical centers: the First Affiliated Hospital of Chongqing Medical University and Southwest Hospital of Army Medical University. Inclusion criteria: (1) No restrictions were placed on sex or age; (2) All cases were confirmed and diagnosed clearly by the hospital; (3) Laboratory data were available for analysis; (4) Patients were not receiving any treatment at the time of diagnosis. Exclusion criteria: (1) Cases with unclear or ambiguous diagnoses; (2) Insufficient laboratory data for analysis; (3) Patients who had received relevant medications prior to diagnosis. This study was approved by the Medical Ethics Committee of Second Affiliated Hospital of Army Medical University (2024–236-01).

### 2.2 Data collection

A total of 54 potential features were collected at the time of patient diagnosis. These parameters included 2 demographic parameters (sex and age) and 52 blood test parameters: WBC, HGB, PLT, MCV, NEUT%, LYM%, MXD%, BASO%, EO%, RBC, EO#, BASO#, HCT, LYM#, MCH, MCHC, MPV, MXD#, NEUT#, PDW, PCT, RDW-CV, RDW-SD, RET%, ALB, GLB, A/G, ALP, ALT, AST, CREA, DBIL, TBIL, GGT, LDH, PT, APTT, TT, Fg, IgA, IgE, IgG, IgM, Igκ, Igλ, EPO, FOL, VB12, SF, FE, IAT, and DAT. The full names of the blood test parameters are provided in the Glossary.

### 2.3 Data preprocessing

Among the 52 blood test parameters included, the missing value ratio of 23 CBC parameters was relatively low, all less than 15%, while the missing values ratio for the other parameters exceeded 30%. First, we performed data cleaning by removing special characters such as “>” and “<” from the parameters in the dataset. To address the missing values, categorical variables were imputed with the mode, while continuous variables were imputed with the median. Subsequently, we standardized the units of all parameters to the international system of units (IS units). Additionally, to eliminate the influence of scales, we applied the StandardScaler to normalize all continuous variables.

### 2.4 Feature selection

To identify the key features for model training, we employed three feature selection methods to create three distinct feature subsets:

1 RF-RFECV (15, 16)

  Using random forest classifier (v1.5.1) with 5-fold cross-validation (CV) and RFECV (v1.5.1), we progressively eliminated less relevant features to obtain an optimal feature subset that enhanced model generalization while improving model interpretability.

2 Light Gradient Boosting Machine (LightGBM) (17, 18)

  We trained a LightGBM model (v4.4.0) and ranked features based on their importance in the tree structure, selecting those with above-average importance values.

3 Information Gain (IG) (19)

  We ranked features based on their entropy-based contribution to the target variable and selected those with information gain values above the average to form the IG feature subset, reducing noise features.

Additionally, we included the complete dataset of all 54 parameters and a clinically relevant subset of 12 parameters (WBC, HGB, PLT, RBC, MCV, NEUT%, LYM%, MXD%, EO%, BASO%, age and sex) validated by three hematology experts. The specific screening method was conducted as follows: from the 23 CBC parameters with minimal missing values, the experts unanimously agreed on 7 core parameters (WBC, HGB, PLT, RBC, MCV, age and sex) for direct inclusion in the model. For the remaining parameters, the experts chose to incorporate NEUT%, LYM%, MXD%, EO%, and BASO% after discussion, as these percentage-based parameters are relative values unaffected by WBC count, making them more comparable across different individuals. This makes them particularly suitable for initial disease screening or classification, especially in the classification of leukemia. Parameters not selected by the expert panel were excluded. Therefore, a total of 5 parameter sets were used for the training of the model.

### 2.5 Model training and optimization

The dataset was randomly divided into a training set and a validation set at a 7:3 ratio. To address the issue of class imbalance, we applied the synthetic minority over-sampling technique (20), which generates synthetic samples to balance the class distribution in the training set. The Optuna framework was utilized for automated hyperparameter tuning through bayesian optimization (21), with an objective function defined as maximizing the coefficient of determination (R^2^) on the validation set.

We evaluated candidate models, including k-nearest neighbor (KNN), random forest (RF), categorical boosting (CatBoost), extreme gradient boosting (XGBoost), LightGBM, alongside two ensemble models: EnMod1, which combines RF, XGBoost, and LightGBM, and EnMod2, which integrates RF, XGBoost, LightGBM, and CatBoost. Model performance was assessed using five-fold cross-validation, with evaluation metrics including precisions, recalls, F1-scores, and area under the curves (AUCs). The initial validation was conducted on the temporally distinct test set (test set_1) and the optimal model demonstrating superior performance was selected for further evaluation. This final model underwent rigorous validation on the multi-center and independent external test set (test set_2), confirming its robust generalization capabilities.

### 2.6 Interpretability

We used SHapley Additive exPlanations (SHAP; version 0.46.0), a widely recognized framework for explaining ML models, to quantify and interpret the contribution of input features. SHAP summary plots visualize the overall impact of each feature, while SHAP dependency plots reveal the relationship between individual features and model predictions. SHAP values quantitatively measure the contribution of each variable to the prediction outcomes, enabling us to rank and evaluate feature importance (22). This analysis enhanced the transparency and interpretability of our ML models, identifying the critical blood laboratory parameters that most influenced the predictions in hematological disease diagnosis.

### 2.7 Clinical diagnostic comparison test

To further evaluate the diagnostic performance and practical application of these models, we conducted a clinical diagnostic comparison test with 100 randomly selected patients from the dataset. The cases were randomly divided into two groups: a 46-parameter group and a 12-parameter group of 50 patients each, all covering 16 disease categories and a group of healthy individuals. Specifically, 4 senior hematologists and 4 junior hematologists were invited for diagnosis. The cases were randomly distributed to each hematologist, and each doctor needed to analyze 20 cases (10 cases in each of the two groups). Finally, the diagnostic accuracy of the two model’s and the two groups of hematologists was evaluated across the two distinct parameter sets.

### 2.8 Development of predictive tools

A web-based calculator using a simplified 12-parameter model has been developed to provide diagnostic support to clinicians and serve as a health assessment platform for users. This platform was employed using the Shiny for Python framework (shiny: version 0.5.0; shinyswatch: version 0.3.1), integrating libraries such as Pandas and Matplotlib for data processing and visualization. In our study, users can input clinical parameters to obtain predicted outcomes for the potential hematological diseases, thereby enhancing the model’s utility and accessibility in clinical diagnosis and patient health assessment.

### 2.9 Statistics method

Data analysis was performed using Python version 3.12.3. For continuous variables, median and quartile ranges (IQR) were used for representation, and comparisons were performed using the Mann-Whitney U test. For discrete variables, they were represented as counts and precentages, and the Chi-square test was used for comparative analysis. During the significance test, the *p* value of parameters was calculated. When the *p* value was less than 0.05, the difference between the two groups of data was considered to be statistically significant.

## 3 Results

### 3.1 Study population

**Figure 1:**
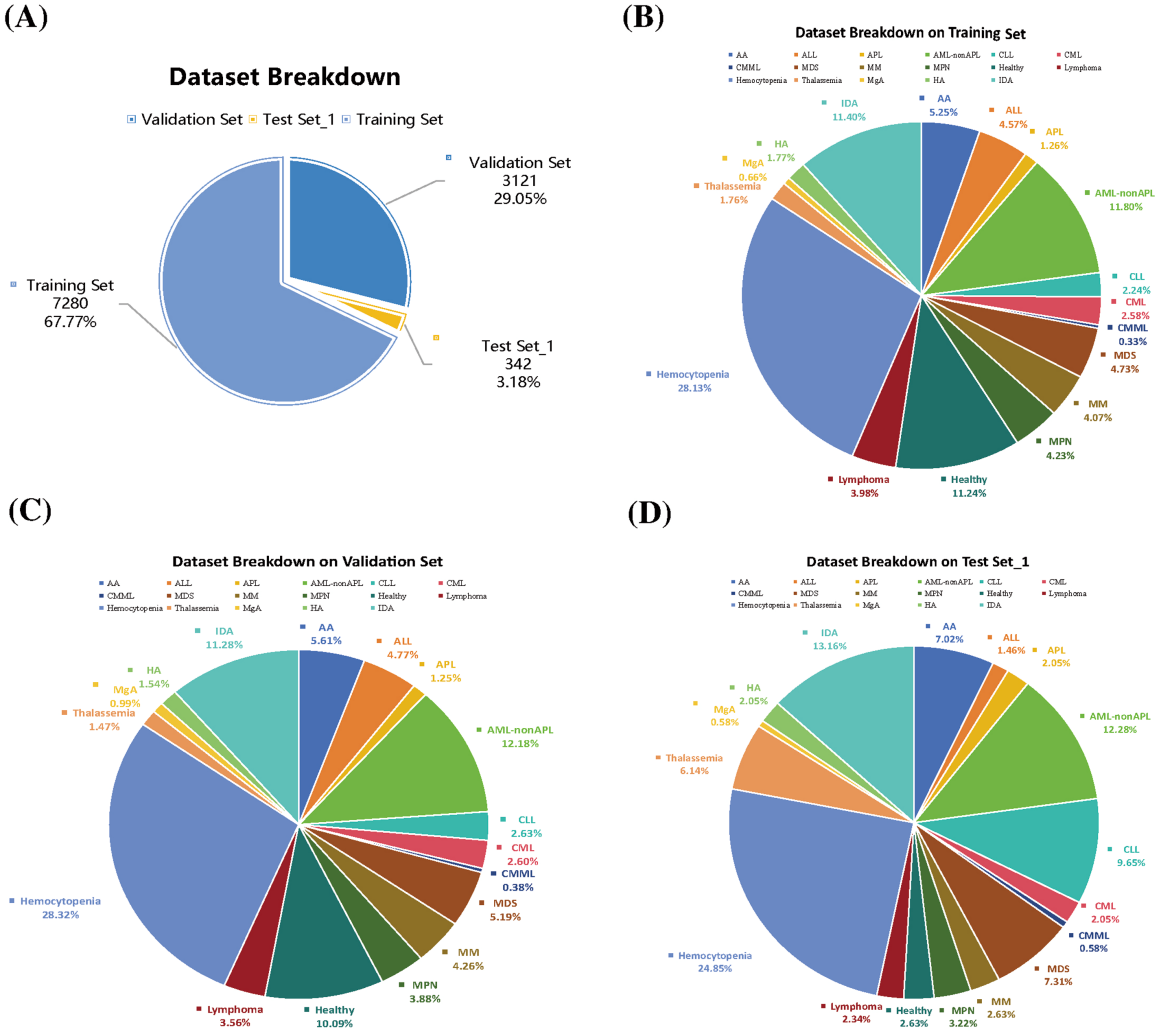
**(A)** Dataset proportions. Disease distribution across datasets of **(B)** Training set. **(C)** Validation set. **(D)** Test set_1.

Our dataset comprised 10,401 patients representing 16 distinct hematological disease types and 1 healthy control group. We analyzed 54 clinical and laboratory parameters for each patient. The dataset was randomly divided into training and validation sets at a 7:3 ratio, consisting of 7,280 and 3,121 cases, respectively. Demographic characteristics were comparable across all sets. The training set had a median age of 46.00 years (IQR: 29.00–57.00) with a male-to-female ratio of 0.85. Similarly, the validation set showed a median age of 46.00 years (IQR: 29.00–58.00) with a male-to-female ratio of 0.88. The test set_1 included 342 patients with a median age of 49.00 years (IQR: 30.00–60.00), comprising 159 males (46.49%) and 183 females (53.51%). The test set_2 with 149 patients had a median age of 55.0 years (IQR: 45.0–69.0) with 78 males (52.35%) and 71 females (47.65%). Supplementary Tables 1, 2 provide the full baseline characteristics of the training/validation sets and test sets. In addition, regarding the dataset distribution, Figure 1A illustrates the overall breakdown of the internal dataset into training, validation, and temporally distinct test sets, demonstrating the proportion of samples allocated to each subset for model development and evaluation. Figures 1B–D further detail the disease category distribution within each of these subsets, respectively. These pie charts show the proportion of each disease category and healthy control samples in the training, validation, and temporally distinct test sets.

### 3.2 Selection of feature parameters

**Figure 2:**
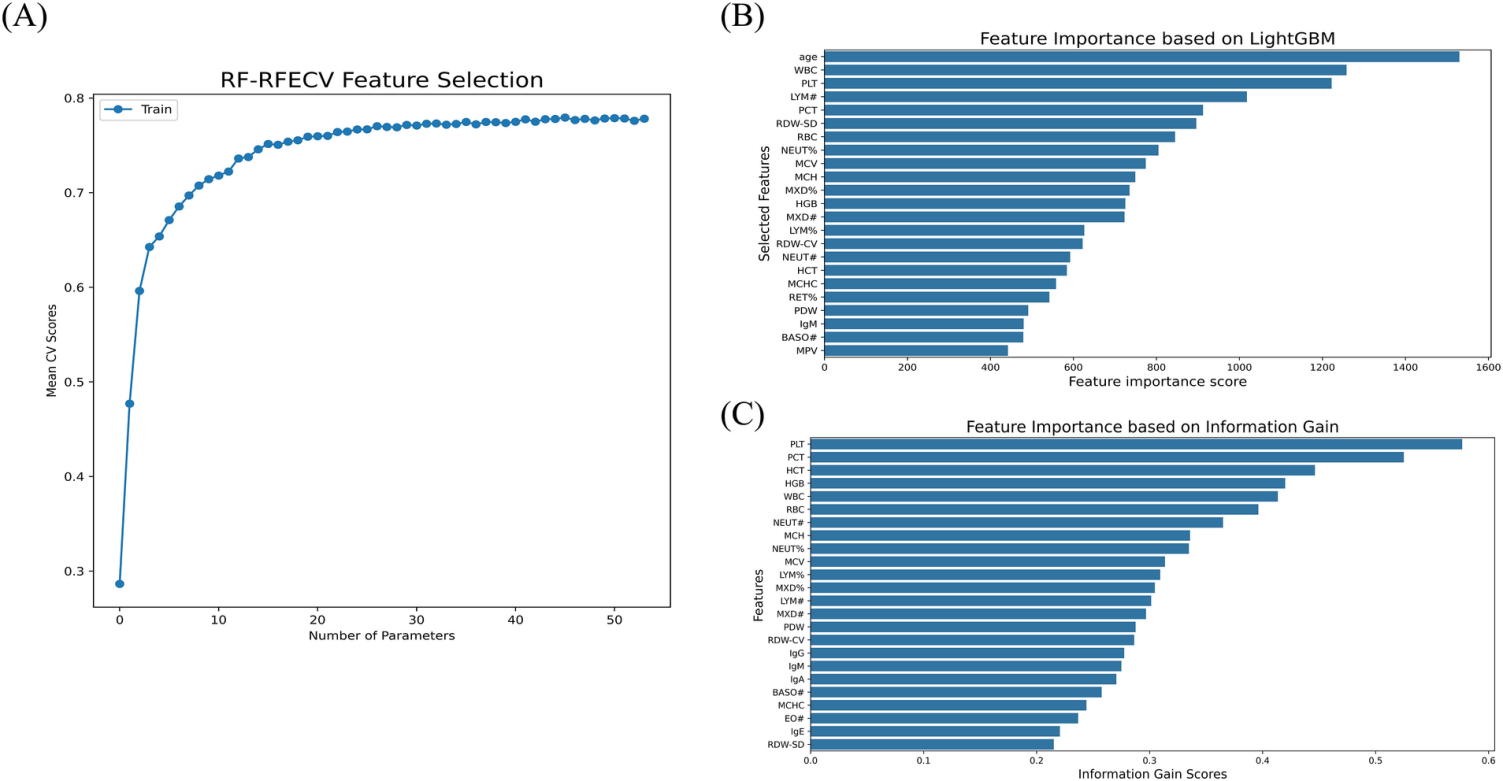
Feature selection. **(A)** RF-RFECV. **(B)** LightGBM. **(C)** IG.

For feature selection, Figure 2A illustrates the relationship between the number of parameters and the mean CV score as determined by the RF-RFECV method. The curve shows a gradual increase in CV scores as more parameters were included, reaching an optimal point at 46 parameters, beyond which the score plateaus. This indicated that 46 parameters were sufficient to capture the essential patterns in the data for optimal model performance. Figures 2B,C show the feature importance scores calculated using the LightGBM algorithm and IG scores, respectively. In total, 23 parameters scored above the average importance threshold with LightGBM, while 24 parameters were selected by IG. Significant parameters like PLT, WBC, and PCT were identified as crucial for the prediction by all methods, although there are slightly different evaluation criteria among them. Detailed information about the selected parameters for each method is shown in Supplementary Table 3.

### 3.3 Model performance and feature importance analysis of the validation set

We assessed the performance of our model using accuracy and AUC as the primary evaluation metrics. On the validation set, the model with 54 parameters achieved a CV score of 0.796, while EnMod1-46, which was developed using the RF-RFECV method to select 46 parameters, achieved a slightly lower CV score of 0.793. Both models demonstrated identical AUC values of 0.979, indicating comparable predictive capabilities. While the performance of the two models was nearly equivalent, EnMod1-46 utilized fewer features, thereby reducing computational costs and minimizing dependence on parameters. Based on this analysis, we selected the 46-parameter EnMod1-46 as our final model. At the same time, EnMod2-12, which was constructed using a simplified parameter set of 12 features, achieved a CV score of 0.739 and an AUC of 0.962. Although its accuracy was lower than that of EnMod1-46, EnMod2-12 offered a practical advantage by requiring fewer and more easily obtainable parameters. Table 1 presents the CV scores and AUCs of different feature subsets across the models.

**Table 1:** The CV scores and AUCs of different models and different parameter sets on the validation set.

Performance Metrics	Model	Light GBM (23 parameters)	IG (24 parameters)	RF-RFECV (46 parameters)	ALL* (54 parameters)	Common* (12 parameters)
CV scores	KNN	0.706	0.699	0.666	0.680	0.661
	RF	0.767	0.750	0.773	0.782	0.735
	CatBoost	0.773	0.751	0.744	0.775	0.734
	XGBoost	0.775	0.754	0.788	0.792	0.731
	LightGBM	0.773	0.757	0.791	0.791	0.726
	Ensemble*	0.779^2^	0.761^2^	0.793^1^	0.796^2^	0.739^2^
AUCs	KNN	0.883	0.866	0.780	0.879	0.856
	RF	0.968	0.968	0.975	0.976	0.959
	CatBoost	0.971	0.967	0.966	0.975	0.959
	XGBoost	0.972	0.968	0.977	0.975	0.952
	LightGBM	0.971	0.966	0.977	0.975	0.957
	Ensemble*	0.974^2^	0.970^2^	0.979^1^	0.979 ^2^	0.962 ^2^

*Ensemble model (1, EnMod1, an ensemble model comprising RF, XGBoost, and LightGBM; 2, EnMod2, an ensemble model comprising RF, XGBoost, LightGBM, and CatBoost). ALL indicates all parameter sets, and Common indicates common parameter sets.

#### 3.3.1 Model EnMod1-46

**Figure 3:**
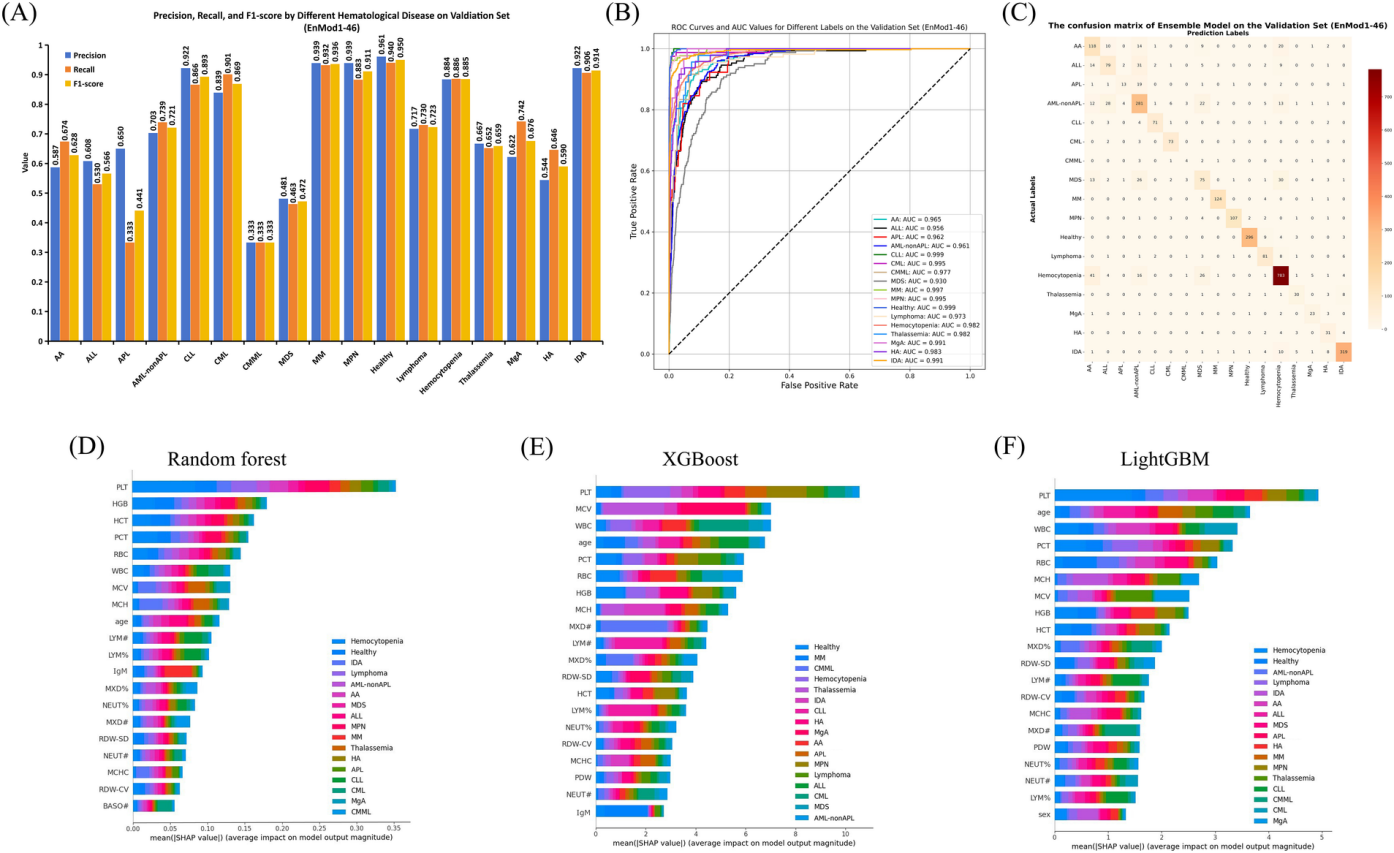
Performance and feature importance analysis of EnMod1-46. **(A)** Classification performance. **(B)** ROC and AUC. **(C)** Confusion matrix. SHAP analysis for EnMod1-46. **(D)** RF. **(E)** XGBoost. **(F)** LightGBM.

The classification performance of the optimal EnMod1-46, constructed using the 46 key feature parameters was evaluated on the validation set, with results presented in Figure 3. Figure 3A displays the precision, recall, and F1-score for each group on the validation set using the EnMod1-46 model. It shows that the model achieved the highest precision and recall for healthy individuals, at 0.961 and 0.940, respectively. The precision and recall of CLL, CML, MM, MPN, hemocytopenia, and IDA were all above 0.800. However, for ALL, APL, and AML-not APL, the precision were slightly lower, at 0.608, 0.650 and 0.703, respectively. The precision of AA, CMML, MDS, and HA were below 0.600, with CMML showing the poorest performance, achieving precision and recall of only 0.333. This might be attributed to the limited sample size of CMML, with only 12 cases available, potentially leading to biased results.

Figure 3B presents the receiver operating characteristic (ROC) curves and AUC values for EnMod1-46 on the validation set. The results showed that the model exhibited excellent performance in diagnosing CLL and healthy group, achieving AUC values of 0.999 for both. In contrast, the AUC value for MDS was the lowest at 0.930. However, excluding MDS, all other groups achieved AUC values exceeding 0.950, demonstrating the model’s robust classification ability across most conditions. Figure 3C presents the confusion matrix for EnMod1-46 on the validation set. As shown in the matrix, the model faced challenges in accurately diagnosing APL, with approximately half of APL cases being misclassified as AML-nonAPL. Additionally, high classification errors were observed among AA, ALL, AML-nonAPL, MDS and Hemocytopenia, suggesting significant overlap in peripheral blood parameters across these conditions. However, the model demonstrated high accuracy in diagnosing CLL and CML, indicating strong differentiation capabilities for these two diseases.

Figures 3D–F present SHAP analysis for EnMod1-46. Eight parameters consistently emerged as top predictors across all three models, including PLT, HGB, WBC, MCV, RBC, PCT, MCH and age. Disease-specific patterns revealed PCT and HGB as primary contributors to Hemocytopenia predictions, while WBC showed strong predictive value for CML and MCV for Thalassemia. Age exhibited particular significance in ALL predictions, and MCH had a notable impact on IDA.

#### 3.3.2 Model EnMod2-12

**Figure 4:**
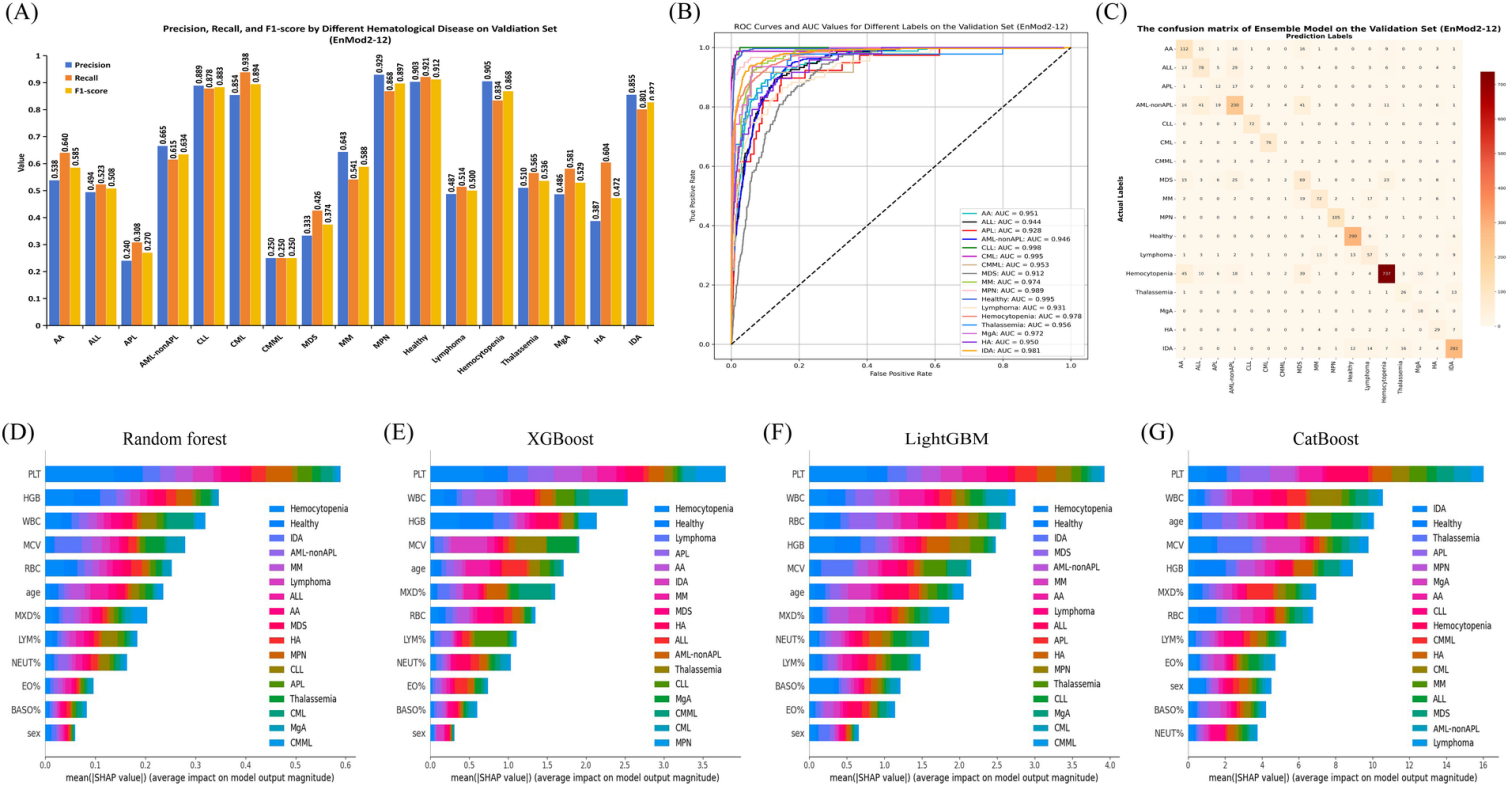
Performance and feature importance analysis of EnMod2-12. **(A)** Classification performance. **(B)** ROC and AUC. **(C)** Confusion matrix. SHAP analysis for EnMod2-12. **(D)** RF. **(E)** XGBoost. **(F)** LightGBM. **(G)** CatBoost.

The classification performance of EnMod2-12 was evaluated on the validation set, with results presented in Figure 4. The figure shows that the classification report results of EnMod2-12 were consistently lower than those of EnMod1-46. Specifically, as demonstrated in Figure 4A, the lowest precision was observed for APL at 0.240, while the highest precision was achieved for MPN at 0.929. In terms of recall, CMML showed the poorest performance at 0.250, while CML achieved the highest recall at 0.938. For F1-score, CMML exhibited the lowest value, while Healthy achieved the highest F1-score at 0.912. In addition, unlike EnMod1-46, which demonstrated precision and recall exceeding 0.800 for seven categories (Healthy, CLL, MPN, CML, Hemocytopenia, IDA, and MM), EnMod2-12 showed a significant decline in precision and recall for MM (Figure 4A).

Figure 4B illustrates the ROC and AUC values for EnMod2-12 on the validation set. The highest AUC value of 0.998 was achieved for CLL, while the AUC value for the healthy group decreased to 0.995 compared to EnMod1-46. MDS still exhibited the lowest AUC value at 0.912, with Lymphoma showing the second lowest AUC value of 0.931.

The confusion matrices for EnMod2-12 on the validation set are presented in Figure 4C. Similar to EnMod1-46, this model struggled with APL diagnosis, frequently misclassifying it as AML-nonAPL. Significant confusion was observed among AA, ALL, AML-nonAPL, MDS, and Hemocytopenia cases, reflecting similarities in their peripheral blood parameters.

Figures 4D–G display corresponding analyses for EnMod2-12. Six of the eight most influential parameters (PLT, WBC, MCV, HGB, RBC, and age) overlapped with those identified in EnMod1-46, maintaining consistent disease-specific associations. For example, PCT retained its strong association with Hemocytopenia predictions across both model architectures, while WBC continued to demonstrate particular importance for CML classification. Based on the data analysis of EnMod1-46 and EnMod2-12, it can be concluded that PLT, WBC, MCV, HGB, RBC and age were the most important characteristic parameters of the prediction models.

### 3.4 Model performance of the test set

On the test set_1, the accuracy of EnMod1-46 was 0.804 and the AUC was 0.964, both of which were better than the 56-parameter models. EnMod2-12 had an accuracy of 0.784 and an AUC of 0.961 (Table 2). This performance advantage persisted in test set_2, the EnMod1-46 attained 0.738 accuracy and 0.973 AUC versus 0.705 accuracy and 0.962 AUC for EnMod2-12. Model diagnostic visualizations are provided in Supplementary Figures: ROC curves with corresponding AUC values for EnMod1-46 (Supplementary Figure S1A, test set_1; Supplementary Figure S2A, test set_2) and EnMod2-12 (Supplementary Figure S1C, test set_1; Supplementary Figure S2C, test set_2), alongside confusion matrices for EnMod1-46 (Supplementary Figure S1B, test set_1; Supplementary Figure S2B, test set_2) and EnMod2-12 (Supplementary Figure S1D, test set_1; Supplementary Figure S2D, test set_2).

**Table 2:** The accuracy and AUC for different models and parameter sets on the test set_1.

Performance Metrics	Model	Light GBM (23 parameters)	IG (24 parameters)	RF-RFECV (46 parameters)	ALL* (54 parameters)	Common* (12 parameters)
Accuracy	KNN	0.690	0.678	0.711	0.693	0.655
	RF	0.784	0.760	0.784	0.781	0.760
	CatBoost	0.763	0.751	0.740	0.787	0.763
	XGBoost	0.769	0.743	0.801	0.778	0.766
	LightGBM	0.781	0.754	0.781	0.775	0.772
	Ensemble*	0.784^2^	0.754^2^	0.804^1^	0.778^2^	0.784^2^
AUC	KNN	0.871	0.856	0.770	0.864	0.888
	RF	0.947	0.939	0.962	0.944	0.947
	CatBoost	0.958	0.948	0.952	0.963	0.954
	XGBoost	0.954	0.945	0.962	0.967	0.936
	LightGBM	0.961	0.942	0.963	0.962	0.956
	Ensemble*	0.960^2^	0.946^2^	0.964^1^	0.962^2^	0.961^2^

*Ensemble model (1, EnMod1, an ensemble model comprising RF, XGBoost, and LightGBM; 2, EnMod2, an ensemble model comprising RF, XGBoost, LightGBM, and CatBoost). ALL indicates all parameter sets, and Common indicates common parameter sets.

### 3.5 Diagnostic agreement analysis

**Figure 5:**
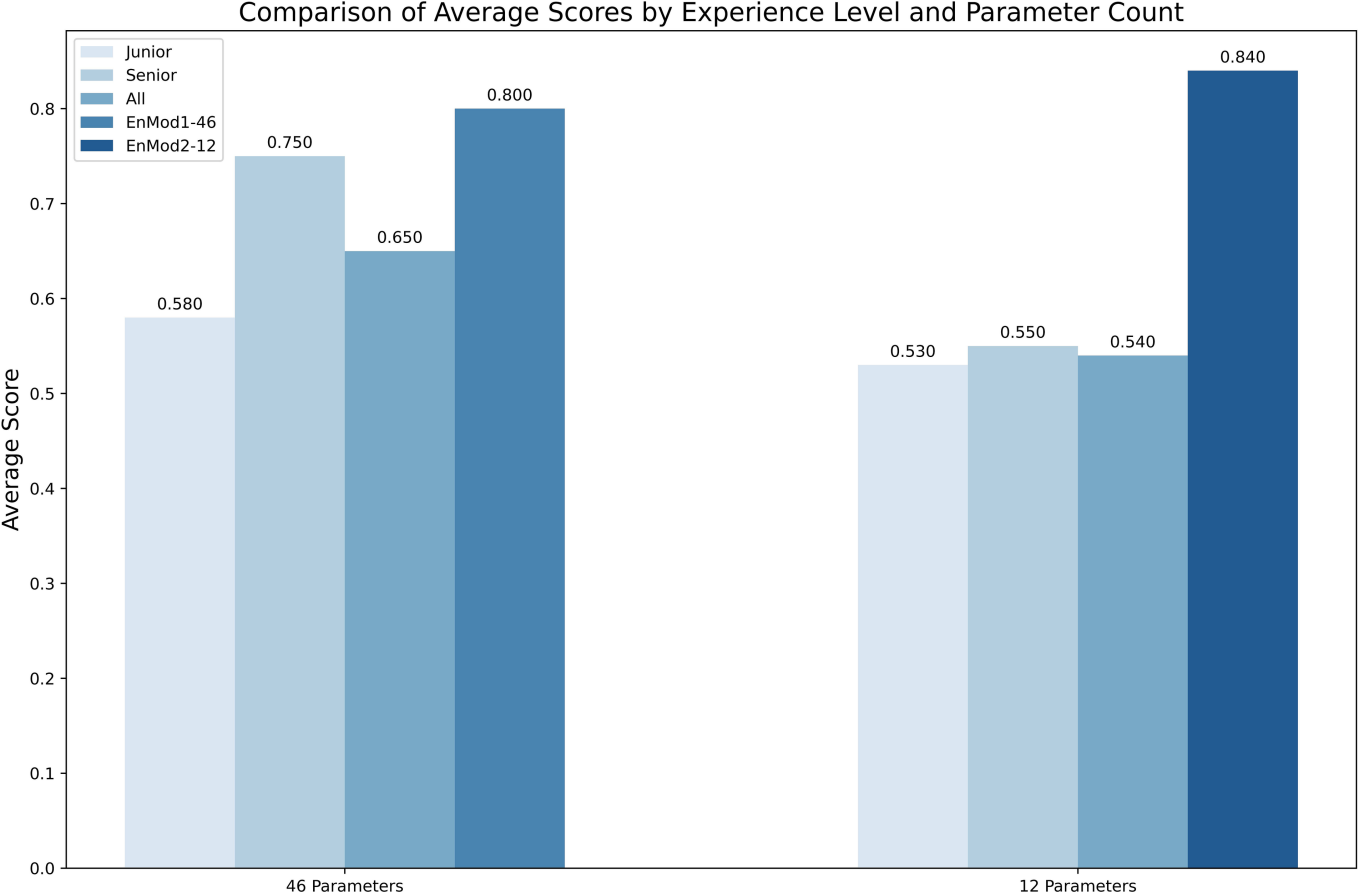
The diagnostic accuracy of clinicians and models using two different parameter sets. Junior, junior hematologist; Senior, senior hematologists; All, both junior and senior hematologists.

Figure 5 presents a comparison of diagnostic accuracy among different groups using different parameter sets. When evaluating diagnoses based on the 12-parameter subset, the EnMod2-12 model demonstrated a diagnostic accuracy of 0.840. In contrast, the mean diagnostic accuracy among five junior hematologists was 0.530, while that of senior hematologists was 0.550. For EnMod1-46, which utilized an expanded 46-parameter dataset, the model achieved a diagnostic accuracy of 0.800. Senior hematologists demonstrated improved performance with this extended parameter set, achieving an accuracy of 0.750, which was slightly lower than the model’s performance. However, junior hematologists showed only a marginal improvement, with their accuracy increasing by just 0.050.

### 3.6 Predictive tool

Considering the challenges that less experienced clinicians may face during the diagnostic process, we have developed an online prediction tool using the EnMod2-12. This practical tool is designed to provide auxiliary diagnostic support for clinical settings, especially for institutions with limited resources. The platform is user-friendly, requiring only the input of 12 key CBC parameters. Among these parameter, “Age” must be entered as an integer and “Gender” requires the user to select the appropriate option. The remaining parameters can accept numeric values. Notably, WBC, HGB, PLT, MCV, and RBC must not be 0, otherwise, the system will issue a warning that the inputs are invalid and request the user to enter the correct value. After inputting the valid values, the user can click the “Predict” button, then the system will rapidly generate precise diagnostic predictions by classifying disease as benign or malignant and providing the corresponding probability for the most possible disease. Therefore, facilitating accurate and timely diagnosis in clinical settings. The system is open to the public and users can use the tool by visiting https://xqidch.shinyapps.io/ham_classification/.

**Figure 6:**
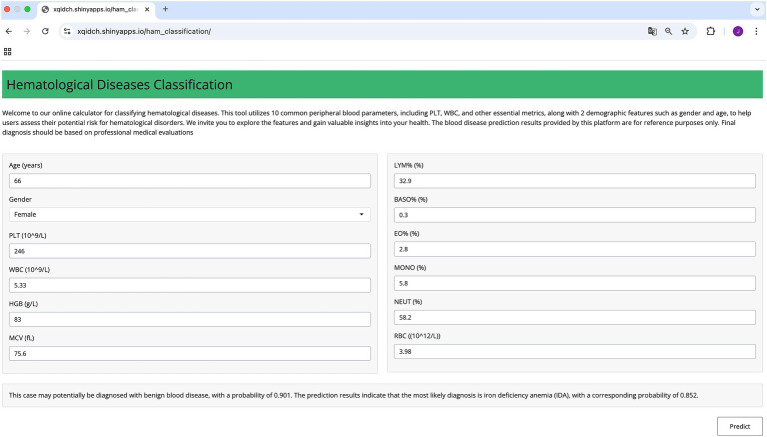
Schematic diagram of the online prediction website.

Figure 6 displays a detailed overview of the online prediction platform using an IDA case as an example. The patient is female, and her age is 66 years old. After entering corresponding data into the platform, the diagnosis revealed a 90.1% likelihood of the patient having a benign hematological disease, and IDA was identified as the most likely disease, with the highest possibility at 85.2%. The diagnostic prediction aligned with the actual clinical diagnosis for this patient.

## 4 Discussion

The heterogeneity of hematological diseases leads to complex correlations between clinical presentation and laboratory parameters. While routine indicators such as CBC and biochemical markers provide valuable diagnostic clues, integrating multi-parameter analyses remains challenging. ML techniques, leveraging their powerful data processing and analysis capabilities, have been widely applied in the diagnosis, prognosis, and prediction of complications in hematologic diseases (23–26). Meanwhile, clinical parameter-based risk prediction models have also demonstrated strong predictive performance in clinical practice (11, 13, 27). ML models based on clinical and laboratory parameters can be cost-effective and efficient for screening hematological diseases, especially malignant ones (28). This study established diagnosis models for common hematological diseases using routine clinical laboratory parameters, achieving high accuracy, stability, and efficiency at low cost, thereby facilitating early diagnosis and screening in clinical settings.

This study developed a predictive model for hematological diseases using peripheral blood parameters. Feature selection is pivotal in ML model construction, as it identifies key features, reduces complexity, and improves generalization. This study employed three feature selection algorithms, with the RF-RFECV algorithm yielding 46 parameters that formed an integrated model, EnMod1-46, showing strong diagnostic performance in all validation and test sets (Tables 1, 2). The advantage of RF-RFECV is that it combines the integration capabilities of random forests with cross-verified feature elimination. This approach better captures complex patterns and feature interactions, which are essential for classifying multiple blood disorders in unbalanced datasets. The integrated model, combining RF, XGBoost, and LightGBM, outperformed single-model approaches by leveraging the strengths of multiple algorithms (29). Additionally, to optimize hyperparameters, we introduced Optuna, an automated optimization framework, simplifying the search process and reducing the time and computational cost of manual tuning (30).

EnMod1-46 achieved accuracies of 0.804 and 0.738 on the test set_1 and the test set_2, respectively, surpassing existing RF-based studies reporting accuracies of 0.57–0.59 (11). While some studies have reported accuracies exceeding 0.90 (13, 14), these models predominantly target single diseases (e.g., specific leukemia subtypes). Additionally, the simplified 12-parameter model, EnMod2-12, achieved test set accuracies of 0.784 (test set_1) and 0.705 (test set_2), offering a practical solution for primary healthcare settings. Despite the reduction in parameters, EnMod2-12 maintained strong predictive capabilities and generalizability, highlighting its potential for applications requiring simpler models. In the validation set, the EnMod1-46 demonstrated high accuracies and recalls for CLL, CML, MM, MPN, hemocytopenia, and IDA (all>0.800). However, performance was lower for CMML (precision: 0.333, recall: 0.333), likely due to insufficient sample size. The confusion matrix analysis demonstrated strong performance in identifying Healthy, CLL, CML, and MM. However, it still faced challenges in distinguishing ALL, APL, and AML-nonAPL.

There were certain differences in the key feature parameters identified by different models, but the overall trend remained consistent in EnMod1-46 and EnMod2-12. SHAP analysis highlighted key parameters contributing to disease prediction, including PLT, WBC, HGB, MCV, RBC, and age. These parameters directly reflect the development of megakaryocytes, myeloid cells, lymphocytes, and red blood cells. Notably, PLT was the most important parameter across all models, consistent with its role in acute leukemia (31). In addition, studies have confirmed that WBC and RBC are key features for identifying leukemia (14). The occurrence of leukemia is due to the abnormal proliferation of leukemic cells in the bone marrow, which often leads to an increase in WBC. This abnormal proliferation of leukemic cells can inhibit normal hematopoiesis, thereby affecting RBC level. HGB is a critical indicator for detecting the presence of anemia, while MCV, MCH, and MCHC are the main parameters for differentiating types of anemia (32). Additionally, different types of hematological diseases have distinct age distributions at onset (33, 34). These key parameters are closely related to the characteristics of disease development. Notably, even without specific parameters such as IgM, the simplified model effectively identified MM (precision: 0.643) on the validation set, demonstrating its validity and reliability. Other studies have demonstrated that clinical parameters such as cytokines, lymphocyte subsets, and cell population data have proven to be highly valuable in the classification and differential diagnosis of hematological diseases (35, 36). In future research, we will incorporate a broader range of clinically valuable parameters to further enhance the predictive scope and accuracy of our models.

Comparative analysis of clinical diagnosis showed that the performance of EnMod2-12 was better than that of hematologists with different experience, indicating that even in the case of fewer parameters, the model can accurately identify complex relationship patterns between different parameters and achieve efficient and accurate predictions. Senior hematologists performed better on 46 parameters, achieving results comparable to EnMod1-46 because hematologists were able to interpret relationships between complex parameters. However, junior hematologists did not improve accuracy when adding parameters. Among them, we also saw that the accuracy rate of EnMod2-12 was better than that of EnMod1-46, which was somewhat different from our research results, which might be caused by differences in data distribution. To facilitate clinical application, we developed a user-friendly web prediction platform based on the simplified EnMod2-12, which can be accessed via the following link: https://xqidch.shinyapps.io/ham_classification/.

Despite the high accuracy achieved in this study, there are still some limitations. First, this single-center retrospective study had incomplete data and insufficient sample sizes for rare diseases, which affected diagnostic accuracy. Second, although this study covered about 80% of hematological disease types, which basically meets clinical needs, further expanding the prediction range will be more clinically valuable. Future studies should increase sample sizes for rare diseases to ensure balanced data and conduct multi-center prospective studies to improve generalizability and clinical application.

## 5 Conclusion

In this study, we developed two high-performance diagnostic models for hematological diseases by employing Optuna for automatic hyperparameter tuning and optimizing the combination of feature algorithms with various ML models. The clinical diagnostic comparison test further validated the significant clinical practical value and diagnostic performance of both models. Additionally, we developed a practical online tool to provide an efficient screening solution and facilitate early diagnosis opportunities for hematological diseases.

## Data Availability

The original contributions presented in the study are included in the article/Supplementary material, further inquiries can be directed to the corresponding authors.

## Glossary

AA: Aplastic Anemia
ALL: Acute Lymphoblastic Leukemia
APL: Acute Promyelocytic Leukemia
AML-nonAPL: Acute Myeloid Leukemia-nonAPL
CLL: Chronic Lymphocytic Leukemia
CML: Chronic Myeloid Leukemia
CMML: Chronic Myelomonocytic Leukemia
MDS: Myelodysplastic Syndrome
MM: Multiple Myeloma
MPN: Myeloproliferative Neoplasm
Hemocytopenia: Immune-related or unclear hemocytopenia
MgA: Megaloblastic Anemia
HA: Hemolytic Anemia
IDA: Iron Deficiency Anemia
WBC: White blood cell count
HCT: Hematocrit
PT: Prothrombin time
HGB: Hemoglobin
MCH: Mean corpuscular hemoglobin
APTT: Activated partial thromboplastin time
PLT: Platelet count
MCHC: Mean corpuscular hemoglobin concentration
Fg: Fibrinogen
MCV: Mean corpuscular volume
MPV: Mean platelet volume
TT: Thrombin time
LYM%: Lymphocyte ratio
PDW: Platelet distribution width
IgA: Immunoglobulin A
LYM#: Lymphocyte count
PCT: Thrombocytocrit
IgE: Immunoglobulin E
BASO%: Basophil ratio
ALB: Albumin
IgG: Immunoglobulin G
BASO#: Basophil count
GLB: Globulin
IgM: Immunoglobulin M
EO%: Eosinophil ratio
ALB/GLB: Albumin/globulin ratio
Igκ: Kappa light chain
EO#: Eosinophil count
ALP: Alkaline phosphatase
Igλ: Lambda light chain
MXD%: Monocyte ratio
ALT: Alanine aminotransferase
EPO: Erythropoietin
MXD#: Monocyte count
AST: Aspartate aminotransferase
FOL: Folic acid
NEUT%: Neutrophil ratio
CREA: Creatinine
VB12: Vitamin B12
NEUT#: Neutrophil count
DBIL: Direct bilirubin
SF: Ferritin
RET%: Reticulocyte ratio
TBIL: Total bilirubin
FE: Serum iron
RBC: Red blood cell count
GGT: Gamma-glutamyl transpeptidase
IAT: Indirect antiglobulin
RDW-CV: Coefficient of variation of red blood cell width
LDH: Lactate dehydrogenase
DAT: Direct antiglobulin
RDW-SD: Standard deviation of red blood cell width.
