# Supplementary material for: Development and application of machine learning models for hematological disease diagnosis using routine laboratory parameters: a user-friendly diagnostic platform
Source: Front Med (Lausanne). 2025 Oct 1;12:1605868. doi: 10.3389/fmed.2025.1605868 (PMC12521225; doi:10.3389/fmed.2025.1605868)
Supplement: Supplementary file 1 [file Data_Sheet_1.docx]

**Supplementary TABLE 1A** The Characteristics of the training and validation sets.

| Variables | Training set  (*n*=7280) | Validation set  (*n*=3121) | *P-*value |
| --- | --- | --- | --- |
| Label, n(%)  AA  ALL  APL  AML-nonAPL  CLL  CML  CMML  MDS  MM  MPN  Healthy  Lymphoma  Hemocytopenia  Thalassemia  MgA  HA  IDA | 382 (5.25%)  333 (4.57%)  92 (1.26%)  859 (11.80%)  163 (2.24%)  188 (2.58%)  24 (0.33%)  344 (4.73%)  296 (4.07%)  308 (4.23%)  818 (11.24%)  290 (3.98%)  2048 (28.13%)  128 (1.76%)  48 (0.66%)  129 (1.77%)  830 (11.4%) | 175 (5.61%)  149 (4.77%)  39 (1.25%)  380 (12.18%)  82 (2.63%)  81 (2.60%)  12 (0.38%)  162 (5.19%)  133 (4.26%)  121 (3.88%)  315 (10.09%)  111 (3.56%)  884 (28.32%)  46 (1.47%)  31 (0.99%)  48 (1.54%)  352 (11.28%) | 0.192 |
| Sex, n(%)  Male  Female | 3339 (45.87%)  3941 (54.13%) | 1459 (46.75%)  1662 (53.25%) | 0.408 |
| Age, median(IQR) | 46.00 (29.00, 57.00) | 46.00 (29.00, 58.00) | 0.332 |
| Clinical measurements, median(IQR) | | | |
| WBC(10^9^/L) | 5.43 (3.41, 7.85) | 5.44 (3.45, 7.89) | 0.421 |
| HGB(g/L) | 101.00(74.00, 131.00) | 99.00 (74.00, 130.00) | 0.141 |
| PLT(10^9^/L) | 93.50 (38.00, 230.00) | 85.00 (35.00, 220.00) | 0.050 |
| MCV(fL) | 92.10 (85.90, 98.00) | 92.10 (86.20, 98.60) | <0.05 |
| LYM%(%) | 31.90 (23.00, 43.10) | 31.90 (22.80, 44.50) | 0.189 |
| LYM#(10^9^/L) | 1.56 (1.08, 2.22) | 1.56 (1.08, 2.23) | 0.821 |
| BASO%(%) | 0.40 (0.20, 0.60) | 0.40 (0.14, 0.60) | 0.276 |
| BASO#(10^9^/L) | 0.02 (0.01, 0.04) | 0.02 (0.01, 0.04) | 0.512 |
| EO%(%) | 1.10 (0.40, 2.20) | 1.00 (0.30, 2.10) | 0.066 |
| EO#(10^9^/L) | 0.06 (0.02, 0.13) | 0.06 (0.02, 0.13) | <0.05 |
| MXD%(%) | 5.70 (4.10, 8.20) | 5.70 (4.10, 8.30) | 0.587 |
| MXD#(10^9^/L) | 0.31 (0.20, 0.47) | 0.31 (0.20, 0.48) | 0.743 |
| NEUT%(%) | 57.60 (43.10, 67.60) | 57.60 (41.20, 67.50) | 0.217 |
| NEUT#(10^9^/L) | 3.01 (1.59, 4.53) | 3.01 (1.56, 4.64) | 0.334 |
| RET%(%) | 1.58 (1.58, 1.58) | 1.58 (1.58, 1.58) | 0.779 |
| RBC(10^12^/L) | 3.75 (2.55, 4.51) | 3.72 (2.52, 4.48) | 0.068 |
| RDW-CV(%) | 14.30 (12.70, 17.00) | 14.40 (12.70, 17.10) | 0.876 |
| RDW-SD(fL) | 49.40 (43.60, 57.90) | 49.40 (43.80, 58.60) | 0.453 |
| HCT(%) | 32.10 (23.70, 40.20) | 31.95 (23.50, 39.82) | 0.197 |
| MCH(pg) | 30.10 (27.90, 32.10) | 30.10 (27.90, 32.10) | 0.208 |
| MCHC(g/L) | 324.00(312.00, 335.00) | 324.00(311.00, 334.00) | 0.409 |
| MPV(fL) | 9.90 (8.80, 11.00) | 9.90 (8.80, 11.00) | 0.258 |
| PDW(%) | 16.40 (15.60, 17.10) | 16.40 (15.60, 17.20) | 0.341 |
| PCT(%) | 0.11 (0.06, 0.21) | 0.11 (0.05, 0.20) | <0.05 |
| ALB(g/L) | 39.05 (39.05, 39.05) | 39.05 (39.05, 39.05) | 0.143 |
| GLB(g/L) | 24.70 (24.70, 24.70) | 24.70 (24.70, 24.70) | 0.197 |
| ALB/GLB(g/L) | 1.58 (1.58, 1.58) | 1.58 (1.58, 1.58) | <0.05 |
| ALP(U/L) | 72.60 (72.60, 72.60) | 72.60 (72.60, 72.60) | 0.316 |
| ALT(IU/L) | 19.20 (19.20, 19.20) | 19.20 (19.20, 19.20) | 0.972 |
| AST(IU/L) | 20.50 (20.50, 20.50) | 20.50 (20.50, 20.50) | 0.589 |
| CREA(mol/L) | 61.00 (61.00, 61.00) | 61.00 (61.00, 61.00) | 0.216 |
| DBIL(µmol/L) | 2.40 (2.40, 2.40) | 2.40 (2.40, 2.40) | 0.701 |
| TBIL(µmol/L) | 12.00 (12.00, 12.00) | 12.00 (12.00, 12.00) | 0.750 |
| GGT(IU/L) | 28.20 (28.20, 28.20) | 28.20 (28.20, 28.20) | 0.850 |
| LDH(IU/L) | 204.70 (204.70, 204.70) | 204.70 (204.70, 204.70) | 0.640 |
| PT(sec) | 11.30 (11.30, 11.30) | 11.30 (11.30, 11.30) | 0.210 |
| APTT(sec) | 30.50 (30.50, 30.50) | 30.50 (30.50, 30.50) | 0.851 |
| Fg(g/L) | 2.60 (2.60, 2.60) | 2.60 (2.60, 2.60) | 0.635 |
| TT(sec) | 16.20 (16.20, 16.20) | 16.20 (16.20, 16.20) | 0.707 |
| IgA(g/L) | 1.87 (1.87, 1.87) | 1.87 (1.87, 1.87) | 0.060 |
| IgE(IU/mL) | 32.00 (32.00, 32.00) | 32.00 (32.00, 32.00) | <0.05 |
| IgG(g/L) | 12.70 (12.70, 12.70) | 12.70 (12.70, 12.70) | 0.193 |
| IgM(g/L) | 1.06 (1.06, 1.06) | 1.06 (1.06, 1.06) | 0.269 |
| Igκ(mg/dL) | 913.00(913.00, 913.00) | 913.00(913.00, 913.00) | 0.795 |
| Igλ(mg/dL) | 520.00(520.00, 520.00) | 520.00(520.00, 520.00) | 0.163 |
| EPO(mIU/L) | 200.00(200.00, 200.00) | 200.00(200.00, 200.00) | 0.726 |
| FOL(ng/mL) | 8.25 (8.25, 8.25) | 8.25 (8.25, 8.25) | 0.118 |
| VB12(pg/mL) | 407.00(407.00, 407.00) | 407.00(407.00, 407.00) | 0.581 |
| SF(ng/mL) | 42.23 (42.23, 42.23) | 42.23 (42.23, 42.23) | 0.400 |
| FE(µmol/L) | 4.00 (4.00, 4.00) | 4.00 (4.00, 4.00) | 0.508 |
| IAT  0.5+  1+  2+  3+  4+  （—） | 7 (0.10%)  12 (0.16%)  20 (0.27%)  12 (0.16%)  8 (0.11%)  7221 (99.19%) | 8 (0.26%)  3 (0.10%)  11 (0.35%)  10 (0.32%)  1 (0.03%)  3088 (98.94%) | 0.164 |
| DAT  0.5+  1+  2+  3+  4+  （—） | 136 (1.87%)  39 (0.54%)  41 (0.56%)  28 (0.38%)  58 (0.80%)  6978 (95.85%) | 54 (1.73%)  25 (0.80%)  21 (0.67%)  10 (0.32%)  26 (0.83%)  2985 (95.64%) | 0.717 |

**Supplementary TABLE 1B** The Characteristics of the test sets.

| Variables | Test set_1  (*n*=342) | Test set_2  (n=149) |
| --- | --- | --- |
| Label, n(%)  AA  ALL  APL  AML-nonAPL  CLL  CML  CMML  MDS  MM  MPN  Healthy  Lymphoma  Hemocytopenia  Thalassemia  MgA  HA  IDA | 24 (7.02%)  5 (1.46%)  7 (2.05%)  42 (12.28%)  33 (9.65%)  7 (2.05%)  2 (0.58%)  25 (7.31%)  9 (2.63%)  11 (3.22%)  9 (2.63%)  8 (2.34%)  85 (24.85%)  21 (6.14%)  2 (0.58%)  7 (2.05%)  45 (13.16%) | 9 (6.04%)  12 (8.05%)  7 (4.70%)  12 (8.05%)  13 (8.72%)  10 (6.71%)  7 (4.70%)  11 (7.38%)  12 (8.05%)  11 (7.38%)  5 (3.36%)  7 (4.70%)  8 (5.37%)  5 (3.36%)  4 (2.68%)  6 (4.03%)  10 (6.71%) |
| Sex, n(%)  Male  Female | 159 (46.49%)  183 (53.51%) | 78 (52.35%)  71 (47.65%) |
| Age, median(IQR) | 49.00 (30.00, 60.00) | 55.00 (45.00, 69.00) |
| Clinical measurements, median(IQR) | | |
| WBC(10^9^/L) | 5.79 (3.65, 9.48) | 6.54 (3.701, 23.39) |
| HGB(g/L) | 102.00 (79.25, 126.75) | 93.00 (69.00, 121.00) |
| PLT(10^9^/L) | 77.00 (29.00, 240.50) | 136.00 (44.00, 213.00) |
| MCV(fL) | 89.00 (78.20, 95.57) | 92.90 (84.90, 98.90) |
| LYM%(%) | 32.80 (22.40, 51.35) | 30.70 (14.40, 46.30) |
| LYM#(10^9^/L) | 1.62 (1.12, 2.61) | 1.72 (1.13, 4.46) |
| BASO%(%) | 0.30 (0.10, 0.50) | 0.20 (0.00, 0.60) |
| BASO#(10^9^/L) | 0.02 (0.01, 0.04) | 0.01 (0.00, 0.05) |
| EO%(%) | 1.00 (0.30, 2.08) | 0.60 (0.00, 1.60) |
| EO#(10^9^/L) | 0.07 (0.02, 0.15) | 0.04 (0.00, 0.13) |
| MXD%(%) | 5.90 (4.62, 8.17) | 6.20 (2.80, 9.00) |
| MXD#(10^9^/L) | 0.34 (0.22, 0.54) | 0.35 (0.21, 0.78) |
| NEUT%(%) | 54.85 (29.02, 65.60) | 52.00 (19.00, 66.50) |
| NEUT#(10^9^/L) | 3.08 (1.63, 4.55) | 3.06 (1.17, 6.05) |
| RET%(%) | 1.58 (1.58, 1.58) | 1.55 (1.55, 1.55) |
| RBC(10^12^/L) | 4.00 (2.64, 4.71) | 3.22 (2.33, 4.25) |
| RDW-CV(%) | 15.00 (13.30, 17.08) | 15.20 (13.50, 18.30) |
| RDW-SD(fL) | 47.45 (42.70, 55.58) | 49.50 (49.50, 49.50) |
| HCT(%) | 32.90 (24.45, 38.78) | 28.20 (21.50, 36.50) |
| MCH(pg) | 29.40 (24.62, 31.4) | 30.70 (28.00, 33.00) |
| MCHC(g/L) | 325.00 (313.00, 33.00) | 330.00 (316.00, 337.40) |
| MPV(fL) | 10.20 (9.43, 11.40) | 9.80 (9.30, 11.10) |
| PDW(%) | 16.20 (15.50, 16.60) | 15.40 (11.40, 16.40) |
| PCT(%) | 0.11 (0.04, 0.23) | 0.14 (0.08, 0.23) |
| ALB(g/L) | 39.05 (39.05, 39.05) | 39.00 (39.00, 42.00) |
| GLB(g/L) | 24.70 (24.70, 24.70) | 24.80 (24.80, 25.10) |
| ALB/GLB(g/L) | 1.58 (1.58, 1.58) | 1.58 (1.58, 1.58) |
| ALT(IU/L) | 19.20 (19.20, 19.20) | 19.40 (15.60, 24.10) |
| AST(IU/L) | 20.50 (20.50, 20.50) | 21.00 (20.40, 32.00) |
| CREA(mol/L) | 61.00 (61.00, 61.00) | 60.90 (60.90, 76.00) |
| DBIL(µmol/L) | 2.40 (2.40, 2.40) | 2.40 (2.40, 3.00) |
| TBIL(µmol/L) | 12.00 (12.00, 12.00) | 12.00 (11.70, 14.70) |
| IgA(g/L) | 1.87 (1.87, 1.87) | 1.87 (1.87, 1.87) |
| IgE(IU/mL) | 32.00 (32.00, 32.00) | 32.50 (32.50, 32.50) |
| IgG(g/L) | 12.70 (12.70, 12.70) | 12.70 (12.70, 12.70) |
| IgM(g/L) | 1.06 (1.06, 1.06) | 1.07 (1.07, 1.07) |
| Igκ(mg/dL) | 913.00 (913.00, 913.00) | 909.00 (909.00, 909.00) |
| Igλ(mg/dL) | 520.00 (520.00, 520.00) | 520.00 (520.00, 520.00) |
| VB12(pg/mL) | 407.00(407.00, 407.00) | 407.00 (407.00, 407.00) |
| ALP(U/L) | 72.60 (72.60, 72.60) | 72.50 (72.50, 98.00) |
| GGT(IU/L) | 28.20 (28.20, 28.20) | 28.00 (26.00, 40.00) |
| Fg(g/L) | 2.60 (2.60, 2.60) | 2.60 (2.60, 2.90) |
| FOL(ng/mL) | 8.25 (8.25, 8.25) | 8.20 (8.20, 8.20) |
| SF(ng/mL) | 42.23 (42.23, 42.23) | 43.51 (43.51, 43.51) |

**Supplementary TABLE 2** The feature parameters of each parameter sets.

| Methods | Numbers | Features |
| --- | --- | --- |
| All | 54 | sex, age, WBC, HGB, PLT, MCV, LYM%, BASO%, EO%, MXD%, NEUT%, RET%, RBC, ALB, GLB, ALB/GLB, ALP, ALT, AST, CREA, DBIL, TBIL, GGT, LDH, PT, APTT, Fg, TT, IgA, IgE, IgG, IgM, Ig-κ, Ig-λ, EPO, FOL, VB12, SF, FE, IAT, DAT, EO#, BASO#, HCT, LYM#, MCH, MCHC, MPV, MXD#, NEUT#, PDW, PCT, RDW-CV, RDW-SD |
| RF-RFECV | 46 | sex, age, WBC, HGB, PLT, MCV, LYM%, BASO%, EO%, MXD%, NEUT%, RET%, RBC, ALB, GLB, ALB/GLB, ALP, ALT, AST, CREA, DBIL, TBIL, GGT, Fg, IgA, IgE, IgG, IgM, Ig-κ, Ig-λ, FOL, VB12, SF, EO#, BASO#, HCT, LYM#, MCH, MCHC, MPV, MXD#, NEUT#, PDW, PCT, RDW-CV, RDW-SD |
| LightGBM | 23 | age, WBC, PLT, LYM#, PCT, RDW-SD, RBC, NEUT%, MCV, MCH, MONO%, HGB, MXD#, LYM%, RDW-CV, NEUT#, HCT, MCHC, RET%, PDW, IgM, BASO#, MPV |
| IG | 24 | WBC, HGB, PLT, MCV, LYM%, MXD%, NEUT%, RBC, IgA, IgE, IgG, IgM, EO#, BASO#, HCT, LYM#, MCH, MCHC, MXD#, NEUT#, PDW, PCT, RDW-CV, RDW-SD |
| Common | 12 | sex, age, WBC, HGB, PLT, MCV, LYM%, BASO%, EO%, MXD%, NEUT%, RBC |

**Supplementary Figure legends**

**FIGURE 1** Classification performance of EnMod1-46 and EnMod2-12 on the test set_1: (A) Confusion matrix of EnMod1-46. (B) ROC and AUC of EnMod1-46. (C) Confusion matrix of EnMod2-12. (D) ROC and AUC of EnMod2-12.

**FIGURE 2** Classification performance of EnMod1-46 and EnMod2-12 on the test set_1: (A) Confusion matrix of EnMod1-46. (B) ROC and AUC of EnMod1-46. (C) Confusion matrix of EnMod2-12. (D) ROC and AUC of EnMod2-12.
